# Supplementary material for: Detection of salivary citrullinated cytokeratin 13 in healthy individuals and patients with rheumatoid arthritis by proteomics analysis
Source: PLoS One. 2022 Mar 23;17(3):e0265687. doi: 10.1371/journal.pone.0265687 (PMC8942274; doi:10.1371/journal.pone.0265687)
Supplement: S1 Table — (DOCX) [file pone.0265687.s001.docx]

**S1 Table. Characteristics of healthy subjects (A, B) and rheumatoid arthritis (RA) patients (C) whose saliva were collected.**

A

| Healthy individual | Age (years) | **S**ex | Smoking history |
| --- | --- | --- | --- |
| 1 | 31 | Male | (-) |
| 2 | 50 | Male | (+) |
| 3 | 44 | Male | (-) |
| 4 | 37 | Male | (-) |
| 5 | 34 | Male | (-) |
| 6 | 33 | Male | (-) |
| 7 | 28 | Male | (-) |
| 8 | 27 | Male | (-) |
| 9 | 27 | Male | (-) |
| 10 | 27 | Male | (-) |

B

| Healthy individual | Age (years) | Sex | Smoking history |
| --- | --- | --- | --- |
| 1 | 31 | Male | (-) |
| 2 | 29 | Female | (-) |
| 3 | 29 | Male | (-) |
| 4 | 30 | Male | (-) |
| 5 | 32 | Male | (-) |
| 6 | 32 | Female | (-) |
| 7 | 31 | Female | (-) |
| 8 | 29 | Female | (-) |
| 9 | 31 | Female | (-) |
| 10 | 29 | Female | (-) |

C

| RA | Age (years) | Sex | CRP (mg/dl) | RF  (IU/ml) | MMP-3  (ng/ml) | Anti-CCP antibody (U/ml) | Smoking history |
| --- | --- | --- | --- | --- | --- | --- | --- |
| 1 | 85 | Female | 1.1 | ＜3.0 | 385.8 | 15.6 | (-) |
| 2 | 65 | Female | 0.04 | 54 | 66.6 | unknown | (-) |
| 3 | 66 | Female | 0.19 | 895.6 | 69.8 | 3.6 | (+) |
| 4 | 58 | Female | 0.13 | 28.3 | 99.1 | 72.7 | (-) |
| 5 | 64 | Male | 0.38 | 73.3 | 170.4 | 73.1 | (+) |
| 6 | 70 | Female | 0.06 | 72.8 | 30.9 | 290 | (+) |
| 7 | 88 | Male | 0.26 | 6.3 | 270.3 | 12.8 | (+) |
| 8 | 49 | Female | 1.79 | 173.6 | 297.6 | 243 | (-) |
| 9 | 69 | Female | 0.03 | 22.3 | 58.6 | 1280 | (-) |
| 10 | 55 | Female | 0.34 | 106.2 | 97.6 | 659 | (+) |
| 11 | 50 | Female | 0.02 | 42.3 | 40.4 | 52.9 | (-) |

A: Healthy individuals used for detection of citrullinated peptides in saliva; B: Healthy individuals used for semi-quantification of Citrullinated Cytokeratin 13 (Cit-CK13) in saliva; C: RA patients used for semi-quantification of Cit-CK13 in saliva.

Smoking history: including current smoking. CRP: C-reactive protein (normal range

0.3 mg/dL or less); RF: Rheumatoid Factor (normal range 15 IU/mL or less); MMP-3:

Matrix Metalloproteinase-3 (normal range: female 17.3-59.7 ng/mL, male 36.9-

121 ng/mL).
