# Supplementary material for: Detection of salivary citrullinated cytokeratin 13 in healthy individuals and patients with rheumatoid arthritis by proteomics analysis
Source: PLoS One. 2022 Mar 23;17(3):e0265687. doi: 10.1371/journal.pone.0265687 (PMC8942274; doi:10.1371/journal.pone.0265687)
Supplement: S2 Table — (DOCX) [file pone.0265687.s002.docx]

**S2 Table. Amino acid sequences of cytokeratin13 peptide identified by matrix 2 assisted laser desorption/ionization time of flight (MALDI-TOF) mass spectrometry.**

| 1 | MSLRLQSSSA | SYGGGFGGGS | CQLGGGRGVS | TCSTRFVSGG | SAGGYGGGVS |
| --- | --- | --- | --- | --- | --- |
| 51 | CGFGGGAGSG | FGGGYGGGLG | GGYGGGLGGG | FGGGFAGGFV | DFGACDGGLL |
| 101 | TGNEKITMQN | LNDRLASYLE | KVRALEEANA | DLEVKIRDWH | LKQSPASPER |
| 151 | DYSPYYKTIE | ELRDKILTAT | IENNRVILEI | DNARLAADDF | RLKYENELAL |
| 201 | RQSVEADING | LRRVLDELTL | SKTDLEMQIE | SLNEELAYMK | KNHEEEMKEF |
| 251 | SNQVVGQVNV | EMDATPGIDL | TRVLAEMREQ | YEAMAERNRR | DAEEWFHTKS |
| 301 | AELNKEVSTN | TAMIQTSKTE | ITELRRTLQG | LEIELQSQLS | MKAGLENTVA |
| 351 | ETECRYALQL | QQIQGLISSI | EAQLSELRSE | MECQNQEYKM | LLDIKTRLEQ |
| 401 | EIATYRSLLE | GQDAKMIGFP | SSAGSVSPRS | TSVTTTSSAS | VTTTSNASGR |
| 451 | RTSDVRRP |  |  |  |  |

Healthy human saliva was separated by two-dimensional electrophoresis, and the part of the gel that matched the spot of citrullinated peptide was cut and analyzed by MALDI-TOF mass spectrometer (Microflex LRF20 [Bruker Daltonics]) and mass spectrometry using peptide mass fingerprinting (Genomine). The underlined part is the amino acid sequence matched to the cytokeratin 13 peptide.
