# Supplementary material for: Detection of salivary citrullinated cytokeratin 13 in healthy individuals and patients with rheumatoid arthritis by proteomics analysis
Source: PLoS One. 2022 Mar 23;17(3):e0265687. doi: 10.1371/journal.pone.0265687 (PMC8942274; doi:10.1371/journal.pone.0265687)
Supplement: S1 Raw images — (PDF) [file pone.0265687.s003.pdf]

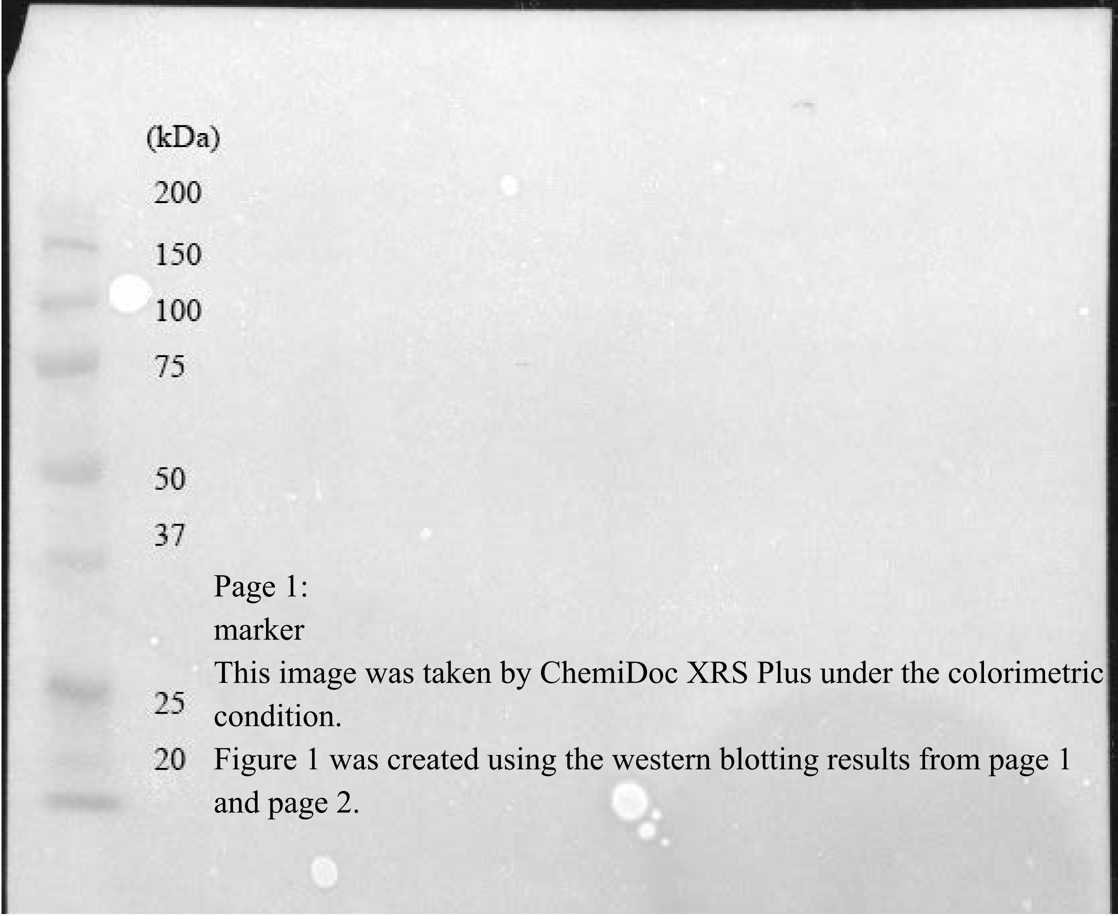

Page 1:

marker

This image was taken by ChemiDoc XRS Plus under the colorimetric condition.

Figure 1 was created using the western blotting results from page 1 and page 2.

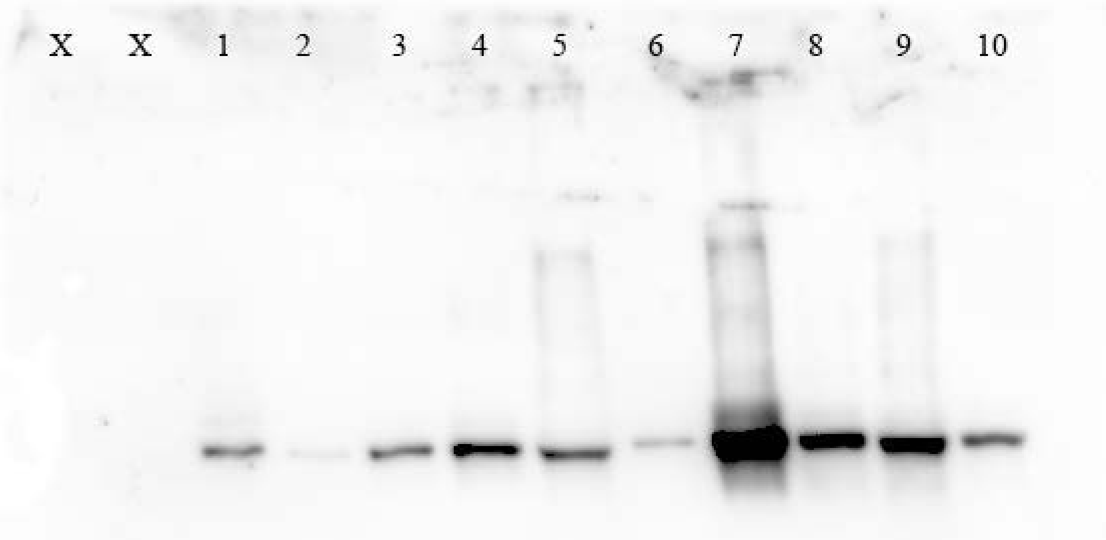

Page 2:
All samples are saliva of healthy humans.
This image was taken under the Chemi (Chemiluminescence) condition of the ChemiDoc XRS Plus.
Figure 1 was created using the western blotting results from page 1 and page 2.

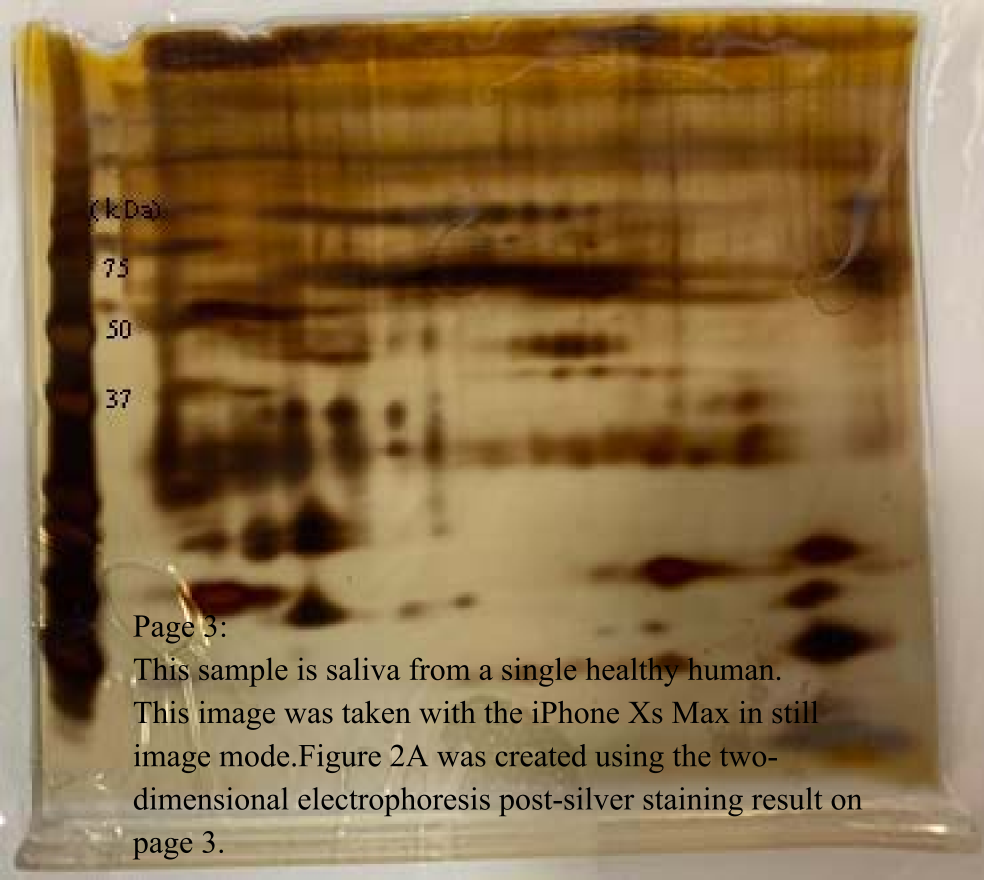

Page 3:

This sample is saliva from a single healthy human. This image was taken with the iPhone Xs Max in still image mode.Figure 2A was created using the two-dimensional electrophoresis post-silver staining result on page 3.

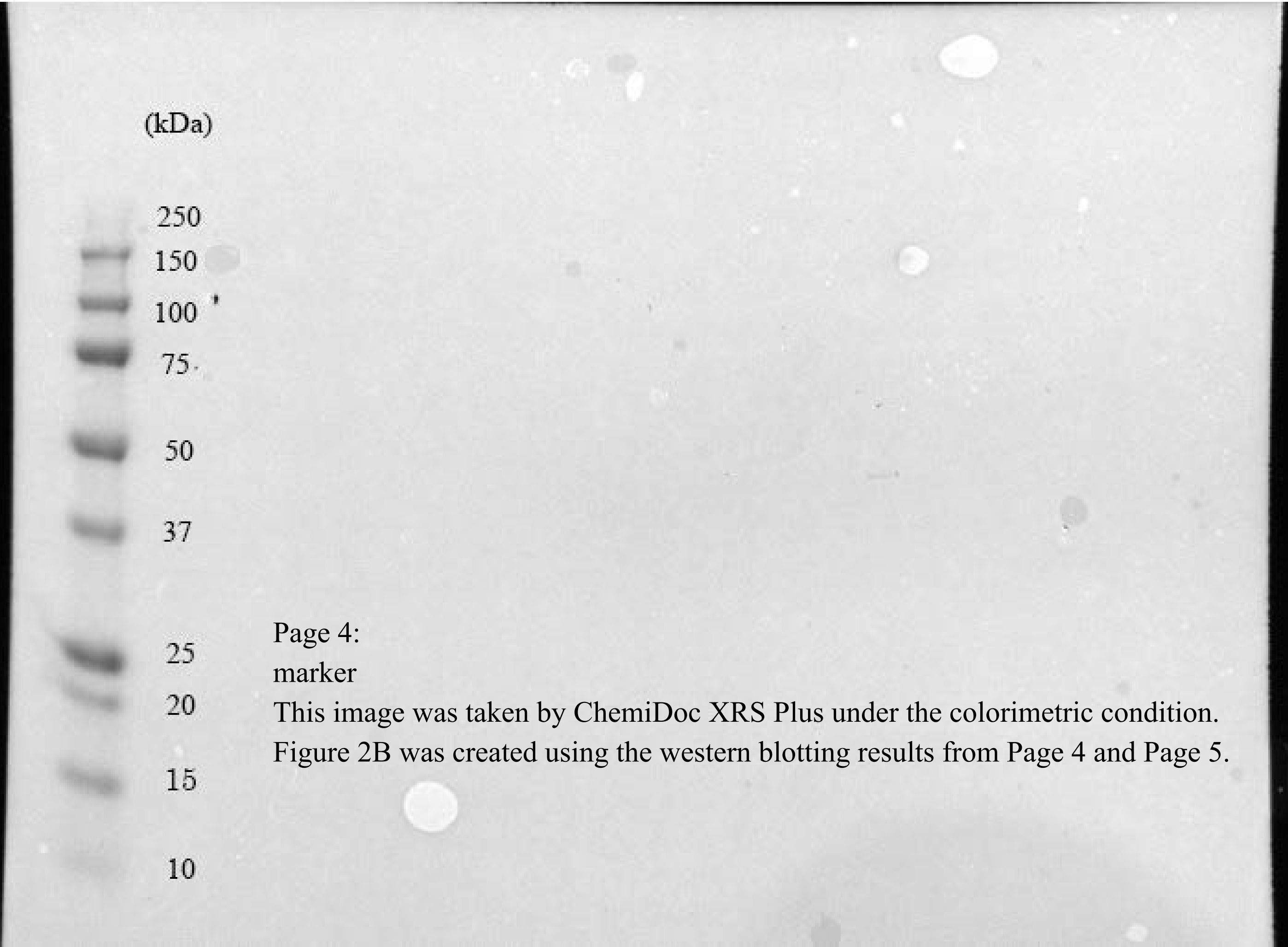

Page 4:

marker

This image was taken by ChemiDoc XRS Plus under the colorimetric condition.

Figure 2B was created using the western blotting results from Page 4 and Page 5.

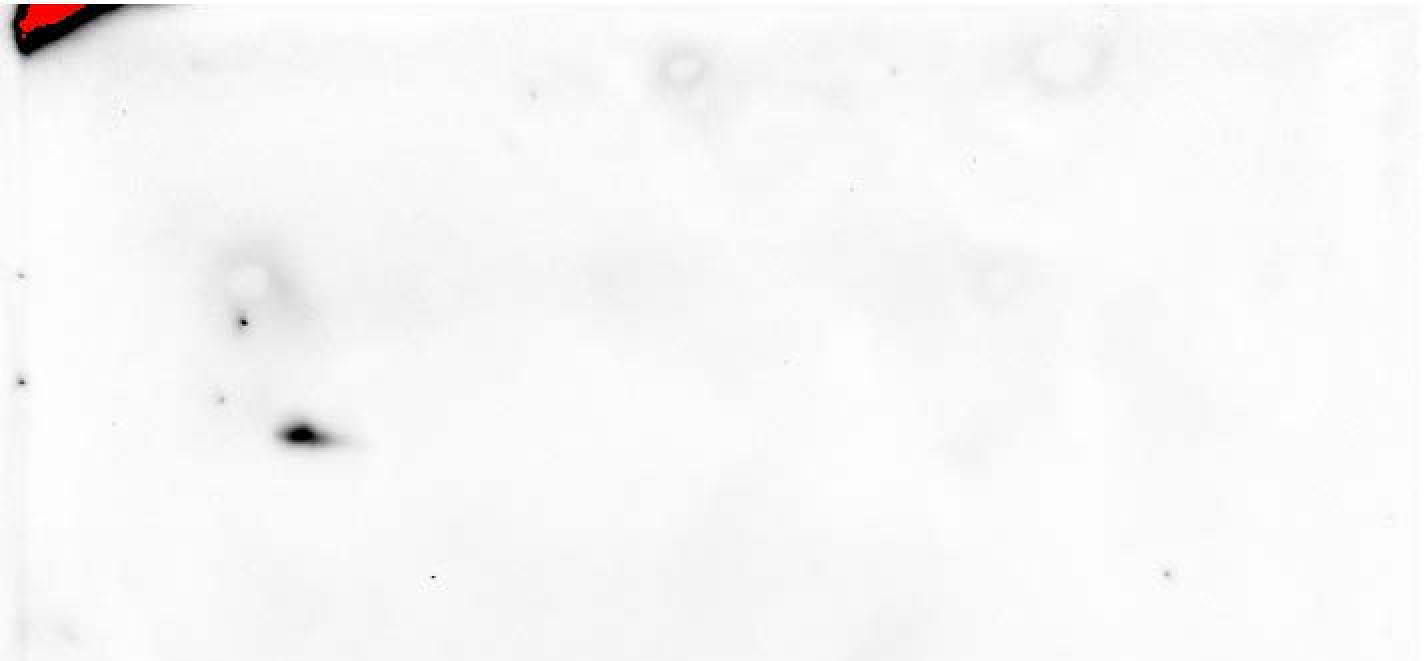

Page 5:

This sample is saliva from a single healthy human.

This image was taken under the Chemi (Chemiluminescence) condition of ChemiDoc XRS Plus.

Figure 2B was created using the western blotting results from Page 4 and Page 5.

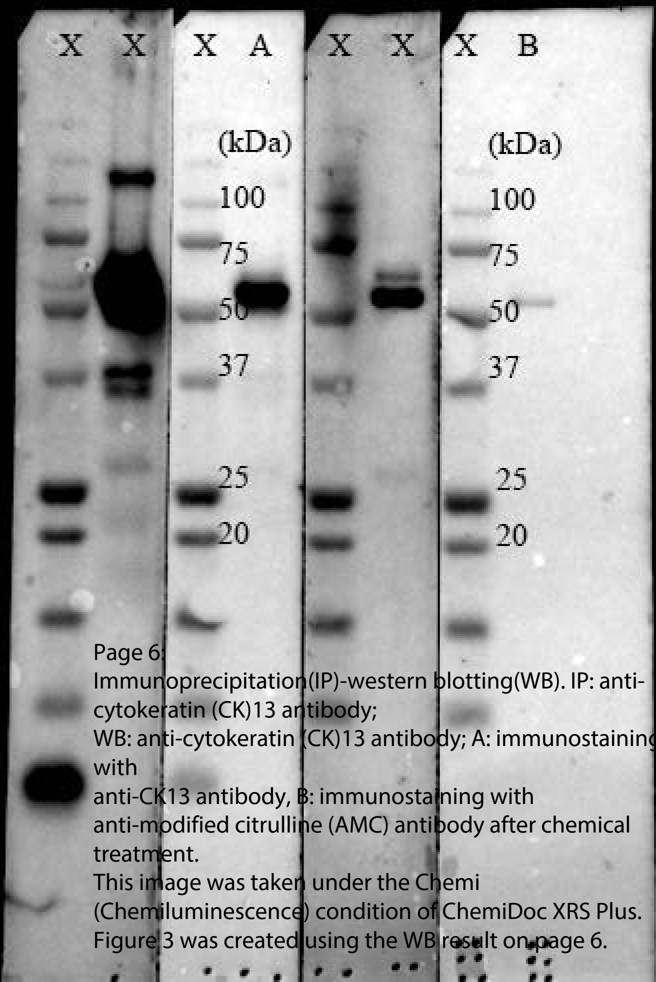

Page 6:
Immunoprecipitation(IP)-western blotting(WB). IP: anti-cytokeratin (CK)13 antibody;
WB: anti-cytokeratin (CK)13 antibody; A: immunostaining with
anti-CK13 antibody, B: immunostaining with
anti-modified citrulline (AMC) antibody after chemical treatment.
This image was taken under the Chemi (Chemiluminescence) condition of ChemiDoc XRS Plus.
Figure 3 was created using the WB result on page 6.

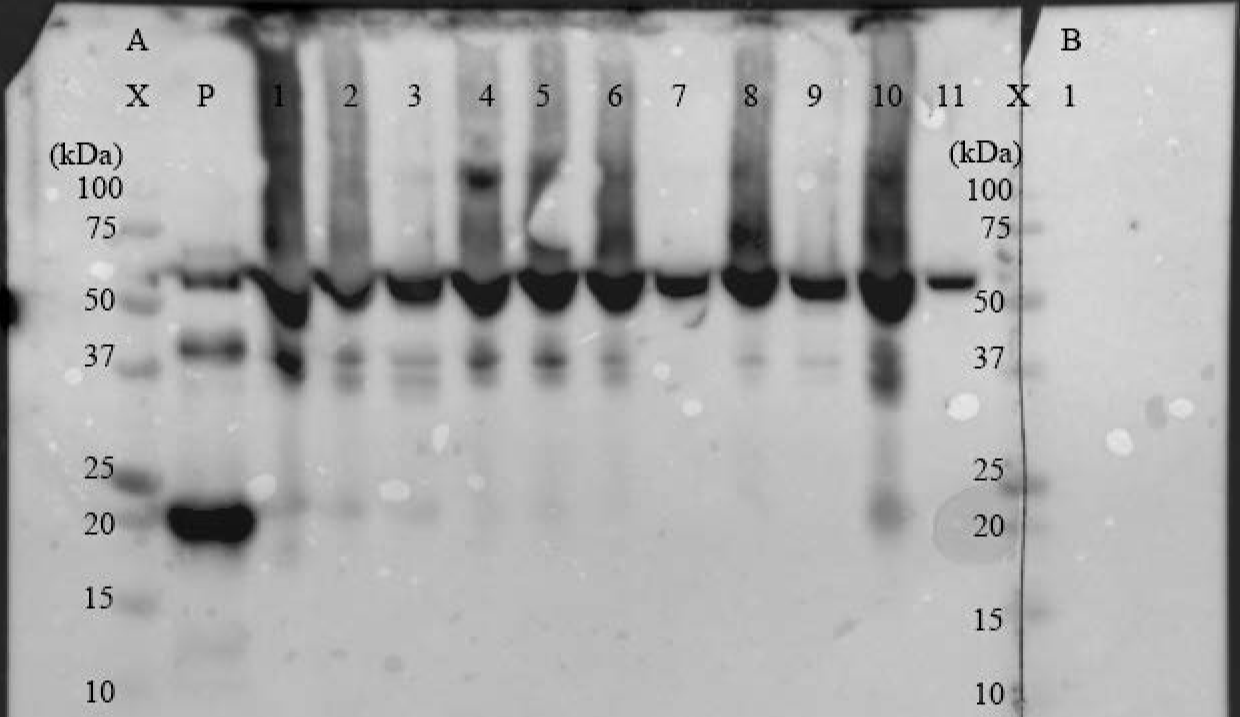

Page7:

(A) P: cytokeratin 13; 1-5: Saliva from patients of rheumatoid arthritis; 6-11: Saliva from healthy individuals; M: marker.

(B) M: marker; 1: Saliva from healthy individuals.

This image was taken under the Chemi (Chemiluminescence) condition of ChemiDoc XRS Plus.

Figure 4 was created using the western blotting result on page 7.

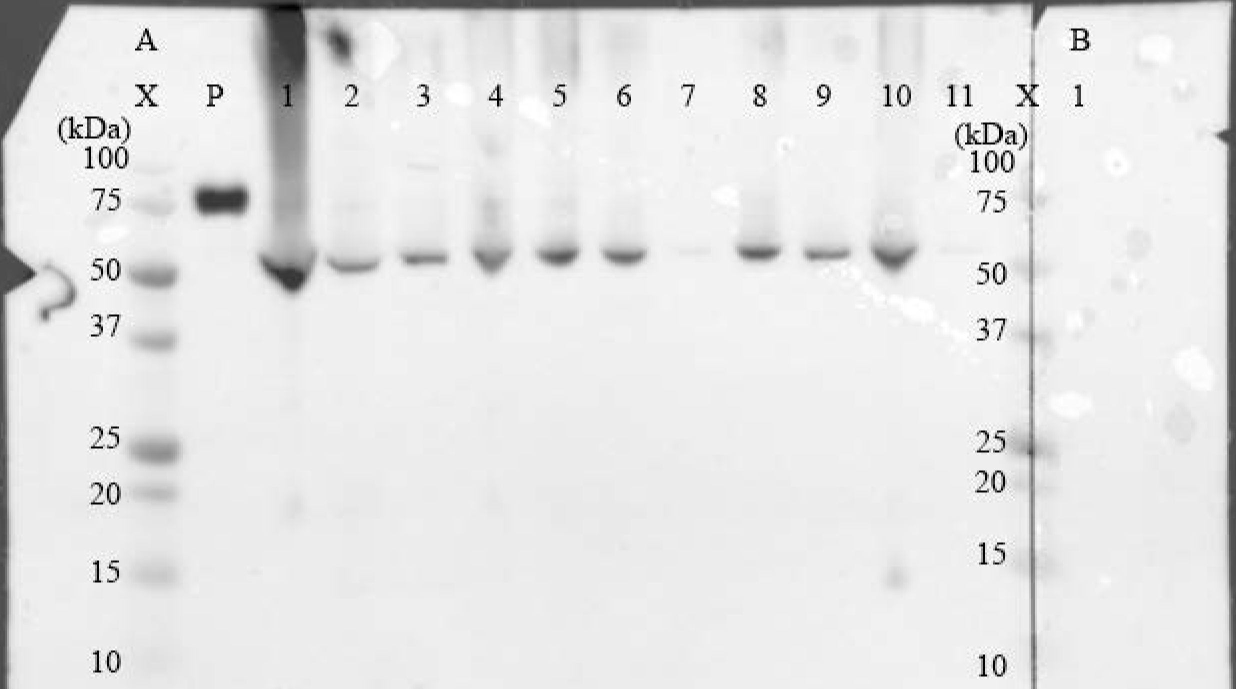

Page 8:
(A) P: citrullinated thrombomodulin; 1-5: Saliva from patients of rheumatoid arthritis; 6-11: Saliva from healthy individuals; M: marker.
(B) M: marker; 1: Saliva from healthy individuals.
This image was taken under the Chemi (Chemiluminescence) condition of ChemiDoc XRS Plus.
Figure 5 was created using the western blotting result on page 8.
